# Multiparametric MRI combined with clinical factors to predict glypican-3 expression of hepatocellular carcinoma

**DOI:** 10.3389/fonc.2023.1142916

**Published:** 2023-11-09

**Authors:** Peijun Liu, Weiqiu Li, Ganbin Qiu, Jincan Chen, Yonghui Liu, Zhongyan Wen, Mei Liang, Yue Zhao

**Affiliations:** ^1^ Department of Radiology, Central People’s Hospital of Zhanjiang, Zhanjiang, China; ^2^ Department of Radiology, The First People’s Hospital of Zhaoqing, Zhaoqing, China

**Keywords:** hepatocellular carcinoma, glypican-3, magnetic resonance imaging, immunotherapy, nomogram

## Abstract

**Objectives:**

The present study aims at establishing a noninvasive and reliable model for the preoperative prediction of glypican 3 (GPC3)-positive hepatocellular carcinoma (HCC) based on multiparametric magnetic resonance imaging (MRI) and clinical indicators.

**Methods:**

As a retrospective study, the subjects included 158 patients from two institutions with surgically-confirmed single HCC who underwent preoperative MRI between 2020 and 2022. The patients, 102 from institution I and 56 from institution II, were assigned to the training and the validation sets, respectively. The association of the clinic-radiological variables with the GPC3 expression was investigated through performing univariable and multivariable logistic regression (LR) analyses. The synthetic minority over-sampling technique (SMOTE) was used to balance the minority group (GPC3-negative HCCs) in the training set, and diagnostic performance was assessed by the area under the curve (AUC) and accuracy. Next, a prediction nomogram was developed and validated for patients with GPC3-positive HCC. The performance of the nomogram was evaluated through examining its calibration and clinical utility.

**Results:**

Based on the results obtained from multivariable analyses, alpha-fetoprotein levels > 20 ng/mL, 75^th^ percentile ADC value < 1.48 ×10^3^ mm^2^/s and R2* value ≥ 38.6 sec^-1^ were found to be the significant independent predictors of GPC3-positive HCC. The SMOTE-LR model based on three features achieved the best predictive performance in the training (AUC, 0.909; accuracy, 83.7%) and validation sets (AUC, 0.829; accuracy, 82.1%) with a good calibration performance and clinical usefulness.

**Conclusions:**

The nomogram combining multiparametric MRI and clinical indicators is found to have satisfactory predictive efficacy for preoperative prediction of GPC3-positive HCC. Accordingly, the proposed method can promote individualized risk stratification and further treatment decisions of HCC patients.

## 1 Introduction

Although the prognosis of HCC has improved with advances in imaging and surgical techniques, the long-term survival outcome remains unsatisfactory due to the high rates of tumor recurrence and metastasis (1, 2). Therefore, specific biomarkers and molecular targets have important clinical significance for early diagnosis and targeted therapies of HCCs. Glypican-3 (GPC3) is an oncofetal glycoprotein expressed in the fetal liver; however, this protein expression is negative in healthy adult livers (3). In contrast, GPC3 is overexpressed in HCCs, which is associated with the occurrence and poor prognosis of HCC (3). It is reported to be involved in cellular growth, migration, differentiation and invasion, indicating its involvement in HCC recurrence and metastasis (4). In addition, GPC3 plays a vital role as an immunotherapeutic target in monoclonal antibody-based HCC therapies (5, 6). Therefore, early identification of GPC3-positive HCC provides excellent clinical value for therapeutic options and prognosis assessment of HCC. Both Wnt and hepatocyte growth factor (HGF) signaling have been reported to promote hepatocarcinogenesis and dissemination of HCC (6). Moreover, as a co-receptor in Wnt and HGF, GPC3 is found to promote the progression of tumor and to be associated with a poor prognosis in HCC (7–9). More specifically, binding to the cell membrane, GPC3 participates in organ morphogenesis by regulating cell proliferation through modulation of Wnt signaling. In addition, it is also involved in migration and motility of HCC cells through heparan sulfate chain-mediated cooperation with the HGF/Met pathway (10). In recent years, it has also received a lot of attention as a new target molecule in immunotherapies. For example, considering the treatment of patients with advanced HCC, Shi D et al. (11) published the first phase I trial of chimeric antigen receptor (CAR)-GPC3 T cells, the results of which confirmed the initial safety and early signs of anti-tumor activity of the cells. In another study, as a GPC3-specific antibody, a novel human monoclonal antibody (32A9) was reported by Liu et al. (10); the antibody was observed to effectively clear GPC3-positive HCC cells *in vitro* and induce HCC xenograft tumor regressions *in vivo*. So, compared with conventional therapies, immunotherapies based on tumor antigen-targeting antibodies as well as immunomodulatory antibodies are considered as emerging approaches in the treatment of HCC (12). Therefore, GPC3 is potentially considered as an effective biomarker for the selection of patients in the immunotherapy of HCC. However, the gold standard of GPC3 evaluation relies on biopsy or surgical resection, which suffers from being invasive and having complications, sampling errors, and time lags. Hence, the development of an accurate non-invasive prediction of GPC3 status is essential and clinically has significant potentials.

Currently, preoperative pathological puncture biopsy can detect the GPC3 expression status of HCC, but it is invasive and may be subject to sampling errors (11); the detection rate of serum GPC3 is limited and lacks satisfactory sensitivity and specificity (6, 11). Therefore, there is an urgent need to develop an accurate and non-invasive method for predicting GPC3 expression. Magnetic resonance imaging (MRI) can be utilized in visualizing a specific target or drug delivery carrier accumulation in tumors (13, 14). Similarly, noninvasive imaging techniques can be used for assessing GPC3 expression as well. Based on previous studies (15), the GPC3 status of HCC can be predicted through applying imaging features (i.e., non-peripheral washout, infiltrative appearance, marked diffusion restriction and iron sparing in solid mass); however, such qualitative features are prone to be contaminated by subjectivity and inter-observer bias. Moreover, compared with qualitative imaging evaluation, radiomics is considered to be a more reliable and accurate assessment of GPC3 status (16–18). Nevertheless, due to the complexity of the process, its poor generalization, and interpretability of the model, its application still faces multiple challenges (19, 20) in clinical practice, which necessitates the urgent development of a feasible and quantitative method capable of predicting the GPC3 status in HCC. Multiparametric MRI techniques has made the non-invasive evaluation a reality due to the high soft-tissue resolution, nonionizing radiation, and a large number of functional imaging approaches. To date, only a few studies have been carried out on the relationship between quantitative MRI features and GPC3 status in HCC. Zhao JT et al. (21), for example, found that lower 75^th^ percentile apparent diffusion coefficient (ADC) value was an independent risk factor associated with GPC3-positive HCC; however, the sensitivity and specificity were unsatisfactory (less than 70%). Moreover, R2* map has been used to measure the content of iron in such liver diseases as fibrosis and iron overload (22, 23). Due to overexpression of transferrin receptor in GPC3-positive HCC patients, the iron content of the tumor increases (24, 25), making the R2* map potentially an excellent way for evaluating GPC3 status in HCC. In another study by Chen R et al. (26), R2* values were observed to be capable of well identifying the status of GPC3 in HCC, with a sensitivity and specificity of about 85%. However, to the best of our knowledge, previous studies have focused merely on MRI techniques, which leaves the question of whether the combination of multiparametric MRI and clinical indicators can improve the diagnostic efficacy of predicting GPC3 status in HCC unanswered. Hence, the present study aims at developing a combined model based on preoperative multiparametric MRI and clinical indicators for predicting GPC3-positive HCC.

## 2 Materials and methods

### 2.1 Study patients

**Figure 1:**
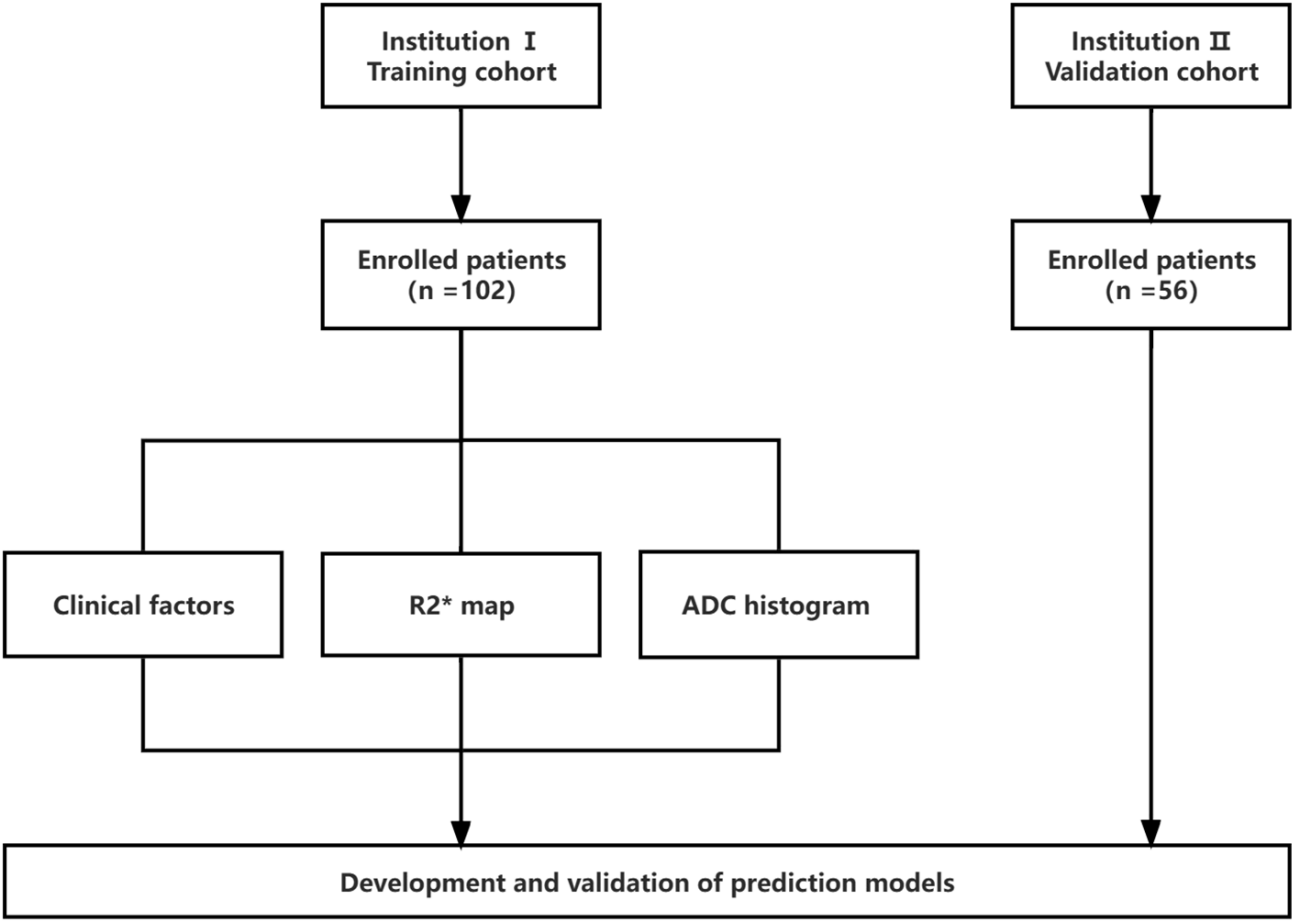
Study flowchart. ADC, apparent diffusion coefficient. R2*, R2-star weighted image.

The present study was approved by the institutional ethics review board. Moreover, due to the retrospective nature of the study, the requirement for informed consent was approved for a waiver. The data were collected from patients at the first people’s hospital of Zhaoqing (Institution I) and central people’s hospital of Zhanjiang (Institution II) between 2020 and 2022. The flow chart of the study is illustrated in **Figure 1**. The inclusion criteria were as follows: (1) age ≥ 18 years; (2) single HCC confirmed pathologically; (3) underwent gadoxetic acid-enhanced MRI within two weeks before surgery, including diffusion weighted imaging (DWI) and R2* map; and (4) complete clinical and pathologic data. The exclusion criteria were as follows: (1) patients receiving alternative treatments, such as radiofrequency ablation or transcatheter arterial chemoembolization (TACE) instead of resection surgery (n = 26); (2) having more than one tumor or satellite nodules (n = 13); (3) occurrence of macrovascular invasion or extrahepatic spreading (n = 7); and (4) inadequate image quality for interpretation (n = 2). Finally, to predict GPC3-positive HCC, 102 patients from institution I served as the training set for the development of the models. The predictive performances of the models were evaluated using a validation set of 56 HCCs from Institution II.

### 2.2 Clinicopathological analyses

Baseline clinical information, including the patients’ demographics, hepatitis B virus infection, and laboratory indicators comprised alpha-fetoprotein (AFP), aspartate aminotransferase, alanine aminotransferase, glutamyl transpeptidase, alkaline phosphatase, albumin, total bilirubin, direct bilirubin, serum creatinine, prothrombin time, neutrophils to lymphocyte ratio, and platelet to lymphocyte ratio.

The histopathological and immunohistochemical analyses were carried out by a liver pathologist with ten years of experience who was not informed of the imaging or clinical outcomes of the study. Information on GPC-3 expression, as documented by another radiologist without knowing the patient’s imaging and clinical data, was retrieved from routine pathological reports as the reference standard in this study. To accurately evaluate the expression of GPC3, we adopted the scoring scale proposed by Takai et al. (27) which took into account positive cell rate and staining intensity. Based on this scoring scale, the positive cell rate was graded from 0 to 3+ as 0 (<5% tumor cells positive), 1+ (5–10% tumor cells positive), 2+ (10–50% tumor cells positive), and 3+ (>50% tumor cells positive). The staining intensity was classified as weak, moderate, and strong staining. In the current study, grade 0 positive cell rate with any staining intensity or grade 1+ positive cell rate with weak staining were regarded as GPC3 negative. Other histopathologic features, including Edmonson-Steiner grade and Ki-67 labeling index, were recorded.

### 2.3 MRI protocol

MRI was performed using a 3.0 T system (SIGNA Premier, GE Healthcare, Waukesha, WI, USA) equipped with a 32-channel abdominal coil (GE Healthcare). The protocol was as follows: an axial breath-hold IDEAL IQ sequence with effective echo time (TE) of 1.0 ms, repetition time (TR) of 6.5 ms, flip angle of 3°, field of view (FOV) of 400×400 mm^2^, bandwidth of 1322 Hz, and slice thickness of 5mm; an axial breathhold T1-weighted 3D fat suppressed spoiled gradient-echo sequence with liver acquisition and volume acceleration and TR of 4.0 ms, TE of 1.5 ms, flip angle of 12°, FOV of 380×380 mm^2^, bandwidth of 762 Hz, and slice thickness of 5 mm; axial T2-weighted fast spin-echo (FSE) sequence with TR of 4255 ms, TE of 72.5 ms, flip angle of 120°, FOV of 360×360 mm^2^, bandwidth of 320 Hz, and slice thickness of 5 mm. Finally, Gadoxetic acid (Primovist; Bayer Schering Pharma, Berlin, Germany) was injected into the cubital vein at a flow rate of 1.0 ml/s and a dose of 0.025 mmol/kg. The T1-weighted 3D fat suppressed spoiled gradient-echo sequence was repeated after the injection of intravenous contrast agent.

### 2.4 Imaging analysis

The qualitative and quantitative MRI features were evaluated by two abdominal radiologists independently (both having 8 years of experience in liver imaging) who were blinded to the patients’ clinical information. Discrepancies were resolved by consensus after re-evaluating the images.

We evaluated the morphological features based on LI-RADS ver.2018 diagnostic algorithm on the Picture Archiving and Communication System (PACS), including *a*) tumor size; *b*) tumor margin; *c*) hemorrhage; *d*) fat component; *e*) target appearance; *f*) mosaic architecture; *g*) washout; *h*) rim arterial phase hyperenhancement (APHE); *i*) peritumoral enhancement; *j*) intratumoral arteries; *k*) enhancing capsule; *l*) peritumoral hypointensity on hepatobiliary phase (HBP).

R2* value was measured on a GE advantage workstation using volume viewer software. In order to avoid necrosis, hemorrhage, fat, and artifacts, the region of interest (ROI) was placed as far as possible in an area with the obvious enhancement of the lesions. The area of ROI was about 1.0~1.5 cm^2^; the lesion was measured three times with the same ROI, the average amounts of which were subsequently calculated.

Apparent diffusion coefficient (ADC) histogram analyses were carried out using the LIFEx software (http://www.lifexsoft.org). Tumor segmentation was performed on the DWI images at 800 s/mm^2^. The image pre-processing stage included the slice selection and gray-level normalization. First, ROI was manually drawn by a radiologist (with 10 years of experience in abdominal imaging) over the whole lesion contour on all slices. Contours were manually drawn for the whole lesion on multiple slices of the axial and multiplanar reconstruction images, with manual adjustments where the initial segmentation was not satisfactory. Accordingly, the final 3D-segmentated volumes were obtained. Second, gray-level normalization, which is known to minimize the effects of brightness and contrast variations on the outcome of histogram analyses, was conducted by scaling the gray-level values to a designated range. Finally, the voxel-based histogram data of ADC were generated for the whole lesion, and the following parameters were calculated: mean, median, skewness, kurtosis, and the percentiles of 25^th^ and 75^th^.

To test the reproducibility of R2* map and ADC histogram parameters, another reader repeated the measurement of R2* values and the whole-tumor histogram analysis in a randomly selected subgroup of 30 study participants.

### 2.5 Data balancing and construction of prediction models

The ratio of GPC3-negative HCCs to GPC3-positive HCCs was 1:4.37 (19 GPC3-negative HCCs and 83 GPC3-positive HCCs) in the training set, revealing a sample imbalance. The synthetic minority over-sampling technique (SMOTE) algorithm was used to balance the minority class in the training set (28), so that the two classes of HCCs were 1:1 (83 GPC3-negative HCCs and 83 GPC3-positive HCCs) in the SMOTE-training set. We developed two prediction models, including logistic regression (LR) in the training set and SMOTE-LR model in the SMOTE-training set.

The performance of prediction model was evaluated by accuracy, sensitivity, specificity, negative-predictive value (NPV), and positive-predictive value (PPV). The area under the receiver operator characteristic curve (AUROC) was employed to evaluate the performance of the GPC3 prediction. The GPC3 predictive performance of the models was further evaluated using the F1 score and the area under the precision-recall curve (AUPRC). Calibration curves were plotted to investigate the model calibration by the Hosmer-Lemeshow test. Decision curve analysis (DCA) was also performed to evaluate the clinical utility of nomogram by quantifying the net benefit under different threshold probabilities.

### 2.6 Statistical analysis

Statistical analysis was conducted with SPSS 23.0 (SPSS, Armonk, NY, USA) and R statistical software (version 3.6.1 R, https://www.r-project.org/). A Student *t*-test (mean ± standard deviation) or Wilcoxon rank-sum test (median, P25 ~ P75) was performed for the continuous variables. The categorical variables were compared using χ^2^ test. Next, the interclass correlation coefficient (ICC) of the quantitative data between the two observers was calculated. To identify the independent predictors of GPC3 expression, multivariable logistic regression analyses were performed. All tests were 2-tailed, and *P* value<0.05 was regarded as statistically significant.

## 3 Results

### 3.1 Clinicopathological features of the training and validation sets

The subjects included 102 patients from institution I, of whom 83 were diagnosed with GPC3-positive HCCs and 19 negative HCCs. In addition, 56 of patients were from institution II, 46 and 10 of whom were diagnosed with GPC3-positive and GPC3-negative, respectively. No significant difference was observed in the distribution of clinicopathologic characteristics for the training and the validation sets (**Table 1**). Based on the results obtained from the univariable analyses in the training cohort, serum AFP levels > 20 ng/mL was observed to be more frequent in GPC3-positive HCCs (*P* = 0.002).

**Table 1:** Baseline clinical characteristics of the training and validation sets.

Characteristics	Training set (n = 102)				Validation set (n = 56)	*p*_Inter_
	Total (n = 102)	GPC3-negative (n = 19)	GPC3-positive (n = 83)	*p*_Intra_		
Age (years)	55 (50 ~ 66)	56 (50 ~ 66)	58 (51 ~ 68)	0.747	52 (45 ~ 63)	0.285
Sex (male)	93 (91.2)	18 (94.7)	75 (90.4)	0.544	53 (94.6)	0.619
HBsAg				0.814		0.096
Negative	18 (17.6)	3 (15.8)	15 (18.1)		11 (19.6)	
Positive	84 (82.4)	16 (84.2)	68 (81.9)		45 (80.4)	
ALT (U/L)	34.00 (25.50 ~ 55.25)	41.60 (27.50 ~ 60.00)	34.50 (20.25 ~ 62.00)	0.486	31.50 (20.00 ~ 54.00)	0.693
AST (U/L)	42.00 (26.00 ~ 48.25)	41.50 (31.25 ~ 49.00)	41.00 (24.00 ~ 48.00)	0.522	41.50 (23.00 ~ 55.25)	0.936
GGT (U/L)	53.00 (37.50 ~ 109.75)	85.00 (47.00 ~ 151.50)	54.00 (39.00 ~ 139.50)	0.249	53.50 (36.75 ~ 120.25)	0.853
ALP (U/L)	83.00 (68.00 ~ 104.00)	87.00 (63.00 ~ 99.50)	83.00 (69.00 ~ 113.00)	0.747	87.00 (69.50 ~ 108.25)	0.457
ALB (g/L)	39.80 (36.58 ~ 42.75)	38.70 (36.70 ~ 41.80)	40.00 (36.50 ~ 43.20)	0.283	38.50 (35.70 ~ 40.70)	0.102
TBIL (µmol/L)	14.82 (11.34 ~ 18.56)	12.59 (9.30 ~ 18.59)	14.84 (11.45 ~ 18.55)	0.437	14.30 (10.60 ~ 17.78)	0.685
DBIL (µmol/L)	4.56 (2.81 ~ 6.41)	5.00 (2.50 ~ 7.28)	4.50 (2.87 ~ 6.57)	0.949	3.35 (2.40 ~ 5.08)	0.057
SCr (U/L)	75.00 (67.00 ~ 86.85)	75.00 (69.00 ~ 87.00)	74.50 (66.70 ~ 80.10)	0.539	76.00 (68.28 ~ 87.30)	0.648
PT (s)	11.80 (11.50 ~ 12.60)	12.30 (11.70 ~ 12.60)	11.80 (11.30 ~ 12.30)	0.106	11.95 (11.40 ~ 12.58)	0.956
NLR	2.47 (1.86 ~ 3.42)	1.85 (1.15 ~ 2.70)	2.05 (1.57 ~ 3.24)	0.197	2.02 (1.59 ~ 3.62)	0.603
PLR	109.94 (71.15 ~ 149.87)	109.15 (62.37 ~ 153.08)	110.73 (72.91 ~ 149.61)	0.901	105.87 (73.30 ~ 166.82)	0.965
AFP (ng/mL)				0.002*		0.583
≤20	48 (47.1)	16 (84.2)	32 (38.6)		47 (83.9)	
>20	54 (52.9)	3 (15.8)	51 (61.4)		9 (16.1)	
Edmondson-Steiner grade				0.920		0.394
I-II	60 (58.8)	11 (57.9)	49 (59.0)		29 (51.8)	
III-IV	42 (41.2)	8 (42.1)	34 (41.0)		27 (48.2)	
Ki-67 labeling index				0.312		0.182
≤15%	38 (37.3)	9 (47.4)	29 (34.9)		15 (26.8)	
>15%	64 (62.7)	10 (52.6)	54 (65.1)		41 (73.2)	

Continuous variables are presented as median (inter-quartile range, IQR). The categorical variables are presented as numbers (percentages). Using univariable association analyses, P_Intra_ is the result of univariate analyses between GPC3-negative and GPC3-positive groups. It also represents whether a significant difference exists between the training and validation datasets.

GPC3, glypican-3; HBsAg, hepatitis B surface antigen; ALT, alanine aminotransferase; AST, aspartate aminotransferase; GGT, glutamyl transpeptidase; ALP, alkaline phosphatase; ALB, albumin; TBIL, total bilirubin; DBIL, direct bilirubin; SCr, serum creatinine; PT, prothrombin time; NLR, neutrophil to lymphocyte ratio; PLR, platelet to lymphocyte ratio; AFP, alpha-fetoprotein.

### 3.2 MRI features of HCCs related to GPC3 in the training set

**Figure 2:**
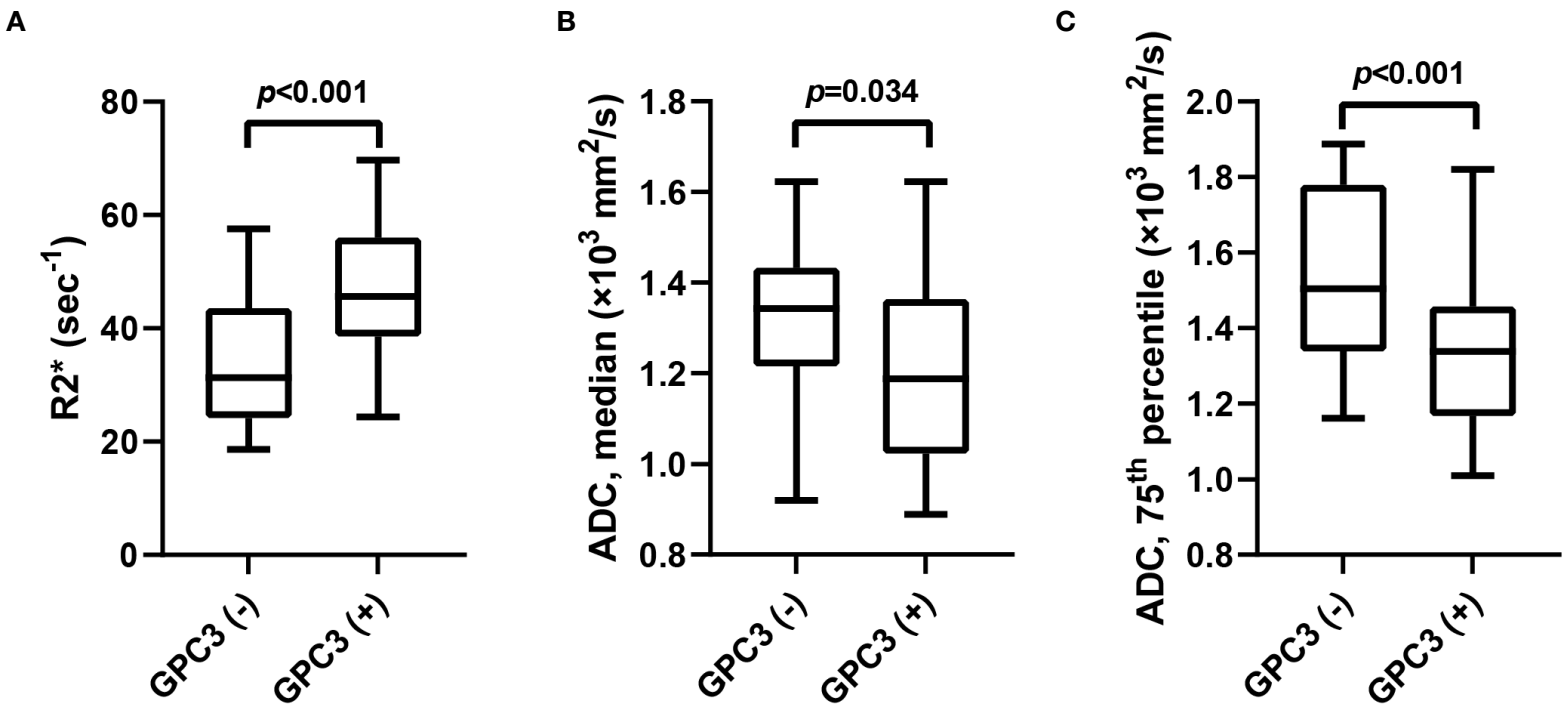
The box-and-whisker plots show the comparison of R2* value **(A)**, median **(B)** and 75th percentile ADC **(C)** values between GPC3-negative and GPC3-positive groups. ADC, apparent diffusion coefficient; GPC3, glypican-3.

The univariable analyses of MRI features in the training set revealed the peritumoral enhancement (χ^2 = ^4.643, *P* = 0.031) to be more frequent in GPC3-positive HCCs. Moreover, as can be seen in **Table 2**; **Figure 2**, the R2* value (*t* = -3.672, *P* < 0.001), the median (*t* = 2.148, *P* =0.034), and the 75^th^ percentile (*t* = 3.585, *P* < 0.001) ADC values in GPC3-positive HCCs were significantly different from those of GPC3-negative HCCs. Furthermore, the optimal cut-off values for the prediction of GPC3-positive HCCs were 38.6 sec^-1^, 1.20 ×10^3^ mm^2^/s, and 1.48 ×10^3^ mm^2^/s for R2*, median and 75^th^ percentile ADC values, respectively. The inter-observer agreements were also found to be excellent for all statistically significant MRI parameters (ICC = 0.869, 95% CI: 0.700 ~ 0.946 for peritumoral enhancement, ICC = 0.878, 95% CI: 0.717 ~ 0.950 for median ADC values, ICC = 0.882, 95% CI: 0.726 ~ 0.952 for 75^th^ percentile ADC values, and ICC = 0.858, 95% CI: 0.731 ~ 0.916 for R2* values).

**Table 2:** Comparison of MRI features between GPC3-negative and GPC3-positive HCCs in training set.

Features	GPC3-negative (n = 19)	GPC3-positive (n = 83)	*p* value
Tumor size	3.6 ± 1.5	4.4 ± 2.1	0.120
Non-smooth tumor margin	12 (63.2)	61 (73.5)	0.368
Hemorrhage	5 (26.3)	25 (30.1)	0.743
Fat component	6 (31.6)	17 (20.5)	0.296
Target appearance	5 (26.3)	22 (26.5)	0.986
Mosaic architecture	4 (21.0)	23 (27.7)	0.622
Non-peripheral washout	13 (68.4)	61 (73.5)	0.655
Rim APHE	7 (36.8)	45 (54.2)	0.172
Peritumoral enhancement	4 (21.1)	40 (48.2)	0.031*
Intratumoral arteries	10 (52.6)	48 (57.8)	0.680
Enhancing capsule	17 (89.5)	59 (71.7)	0.097
Peritumoral hypointensity on HBP	4 (21.1)	36 (43.4)	0.072
R2* (sec^-1^)	34.48 ± 11.20	46.29 ± 12.94	<0.001*
ADC, mean (×10^3^ mm^2^/s)	1.19 ± 0.24	1.23 ± 0.19	0.438
ADC, median (×10^3^ mm^2^/s)	1.32 ± 0.18	1.22 ± 0.19	0.034*
ADC, skewness (×10^3^ mm^2^/s)	0.37 (-0.02 ~ 0.51)	0.28 (0.02 ~ 0.56)	0.935
ADC, kurtosis (×10^3^ mm^2^/s)	1.22 (0.45 ~ 2.34)	1.35 (0.34 ~ 2.73)	0.689
ADC, 25^th^ percentile (×10^3^ mm^2^/s)	1.01 ± 0.22	1.02 ± 0.23	0.898
ADC, 75^th^ percentile (×10^3^ mm^2^/s)	1.52 ± 0.26	1.33 ± 0.20	<0.001*

*p<0.05. Except where indicated, data are numbers of patients, with percentages in parentheses.

HCC, hepatocellular carcinoma; GPC3, glypican-3; APHE, arterial phase hyperenhancement; HBP, hepatobiliary phase; ADC, apparent diffusion coefficient.

**Figure 3:**
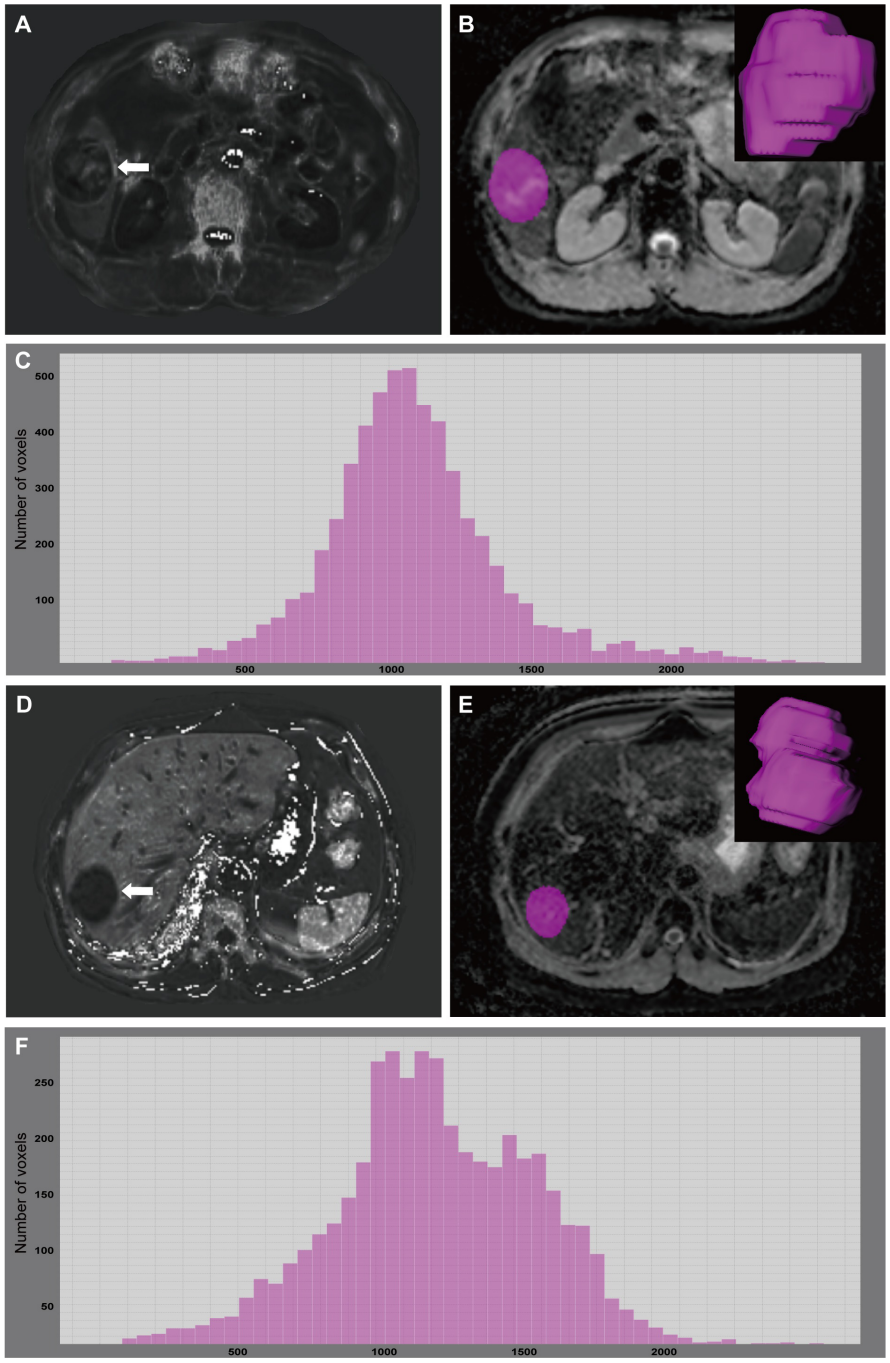
A 49-year-old male with GPC3-positive HCC. A mass (arrow) was located in the hepatic segment V with subtly hyperintense in the R2* map **(A)**. R2* value of the tumor was 55.73 sec^-1^. The tumor was segmented on ADC map and the corresponding volume-rendering image **(B)**. Based on the corresponding histogram **(C)** of the whole-tumor ADC map, the lower ADC values were the more frequently seen ones. 75^th^ percentile ADC value of the tumor was 1.276×10^3^ mm^2^/s. A 67-year-old male with GPC3-negative HCC. A mass (arrow) was located in the hepatic segment VII with hypointense in the R2* map **(D)**. R2* value of the tumor was 24.38 sec^-1^. The tumor was segmented on ADC map and the corresponding volume-rendering image **(E)**. Based on the corresponding histogram **(F)** of the whole-tumor ADC map, the higher ADC values were the more frequently seen ones. 75^th^ percentile ADC value of the tumor was 1.496×10^3^ mm^2^/s. GPC3, glypican-3; HCC, hepatocellular carcinoma; ADC, apparent diffusion coefficient.

With regards to the multivariable analyses, serum AFP levels of > 20 ng/mL (OR = 14.686 [95% CI: 1.829 ~ 117.906], *P* = 0.011), R2* value ≥ 38.6 sec^-1^ (OR = 13.337 [95% CI: 2.665 ~ 66.751], *P* = 0.002) and 75^th^ percentile ADC value < 1.48 ×10^3^ mm^2^/s (OR = 4.900 [95% CI: 1.114 ~ 21.544], *P* = 0.035) were found to be independent significant variables associated with GPC3-positive HCCs (**Table 3**; **Figure 3**). Furthermore, the significance level of Hosmer-Lemeshow test was obtained to be 0.117, suggesting an acceptable goodness-of-fit for the model.

**Table 3:** Univariable and multivariable logistic regression of clinical and MRI features for GPC3-positive HCCs.

Characteristics	Univariable		Multivariable	
	OR (95% CI)	*p* value	OR (95% CI)	*p* value
AFP > 20 ng/mL	8.333 (2.247 ~ 30.901)	0.002*	14.686 (1.829 ~ 117.906)	0.011*
Enhancing capsule	0.289 (0.062 ~ 1.349)	0.114		
Peritumoral enhancement	3.488 (1.068 ~ 11.398)	0.039*		
Peritumoral hypointensity on HBP	2.872 (0.878 ~ 9.397)	0.081		
R2* ≥ 38.6 sec^-1^	7.764 (2.504 ~ 24.069)	<0.001*	13.337 (2.665 ~ 66.751)	0.002*
ADC, median < 1.20 ×10^3^ mm^2^/s	4.031 (1.234 ~ 13.172)	0.021*		
ADC, 75^th^ percentile < 1.48 ×10^3^ mm^2^/s	6.777 (2.309 ~ 19.891)	<0.001*	4.900 (1.114 ~ 21.544)	0.035*

*p<0.05. HCC, hepatocellular carcinoma; GPC3, glypican-3; OR, odds ratio; CI, confident interval; AFP, alpha-fetoprotein; HBP, hepatobiliary phase; ADC, apparent diffusion coefficient.

### 3.3 Performance of the prediction models

Due to the high proportion of GPC3-positive HCCs, we observed high sensitivity (0.831) and PPV (0.932) but obviously low specificity (0.737) and NPV (0.500) of the LR model in training set. In contrast, although the sensitivity (0.807) and PPV (0.905) of SMOTE-LR model in training set declined, the specificity (0.915) and NPV (0.826) improved greatly (**Table 4**).

**Table 4:** Performance of GPC3-positive HCCs prediction models in the training set.

Models		ACC	SEN	SPE	PPV	NPV	AUROC	AUPRC	F1 Score
AFP > 20 ng/mL	Training	0.657	0.614	0.842	0.944	0.333	0.728	0.919	0.744
	Internal test	0.679	0.696	0.600	0.889	0.300	0.648	0.887	0.781
	External test	0.678	0.717	0.500	0.868	0.278	0.609	0.865	0.785
R2* > 38.6 sec^-1^	Training	0.794	0.807	0.737	0.931	0.467	0.772	0.889	0.864
	Internal test	0.768	0.783	0.700	0.923	0.412	0.741	0.863	0.847
	External test	0.678	0.696	0.600	0.889	0.300	0.648	0.846	0.781
ADC, 75^th^ percentile < 1.48 ×10^3^ mm^2^/s	Training	0.745	0.735	0.790	0.938	0.405	0.762	0.922	0.824
	Internal test	0.732	0.718	0.800	0.943	0.381	0.759	0.901	0.815
	External test	0.720	0.701	0.760	0.921	0.389	0.730	0.883	0.796
LR	Training	0.814	0.831	0.737	0.932	0.500	0.851	0.943	0.878
	Internal test	0.821	0.848	0.700	0.928	0.500	0.834	0.923	0.886
	External test	0.804	0.827	0.700	0.921	0.467	0.769	0.905	0.871
SMOTE-LR	Training	0.861	0.807	0.915	0.905	0.826	0.911	0.973	0.853
	Internal test	0.847	0.796	0.887	0.864	0.801	0.887	0.954	0.829
	External test	0.832	0.765	0.851	0.832	0.787	0.843	0.925	0.797
SMOTE-LR*	Training	0.837	0.817	0.895	0.869	0.615	0.909	0.963	0.842
	Internal test	0.834	0.824	0.854	0.847	0.584	0.854	0.937	0.835
	External test	0.821	0.848	0.700	0.817	0.500	0.829	0.916	0.832

* represents the SMOTE-LR model after omitting the synthetic samples in the SMOTE-training set.

HCC, hepatocellular carcinoma; GPC3, glypican-3; AFP, alpha-fetoprotein; ADC, apparent diffusion coefficient; ACC, accuracy; SEN, sensitivity; SPE, specificity; PPV, positive predictive value; NPV, negative predictive value; AUROC, area under the receiver operating characteristic curve; AUPRC, area under the precision-recall curve.

After omitting the synthetic samples in the SMOTE-training set, the AUC of the SMOTE-LR model was 0.909 (95% confidence interval: 0.765 ~ 0.984). Moreover, the accuracy, sensitivity, specificity, PPV and NPV were found to be 0.837, 0.807, 0.895, 0.869 and 0.615, respectively. Furthermore, the precision-recall analyses further revealed the AUPRC of 0.963 and F1 score of 0.899 for the SMOTE-LR model. On the externally validated dataset, these measures were 0.829 (95% CI: 0.765 ~ 0.925), 0.821, 0.848, 0.700, 0.817, 0.500, 0.916 and 0.832, respectively.

**Figure 4:**
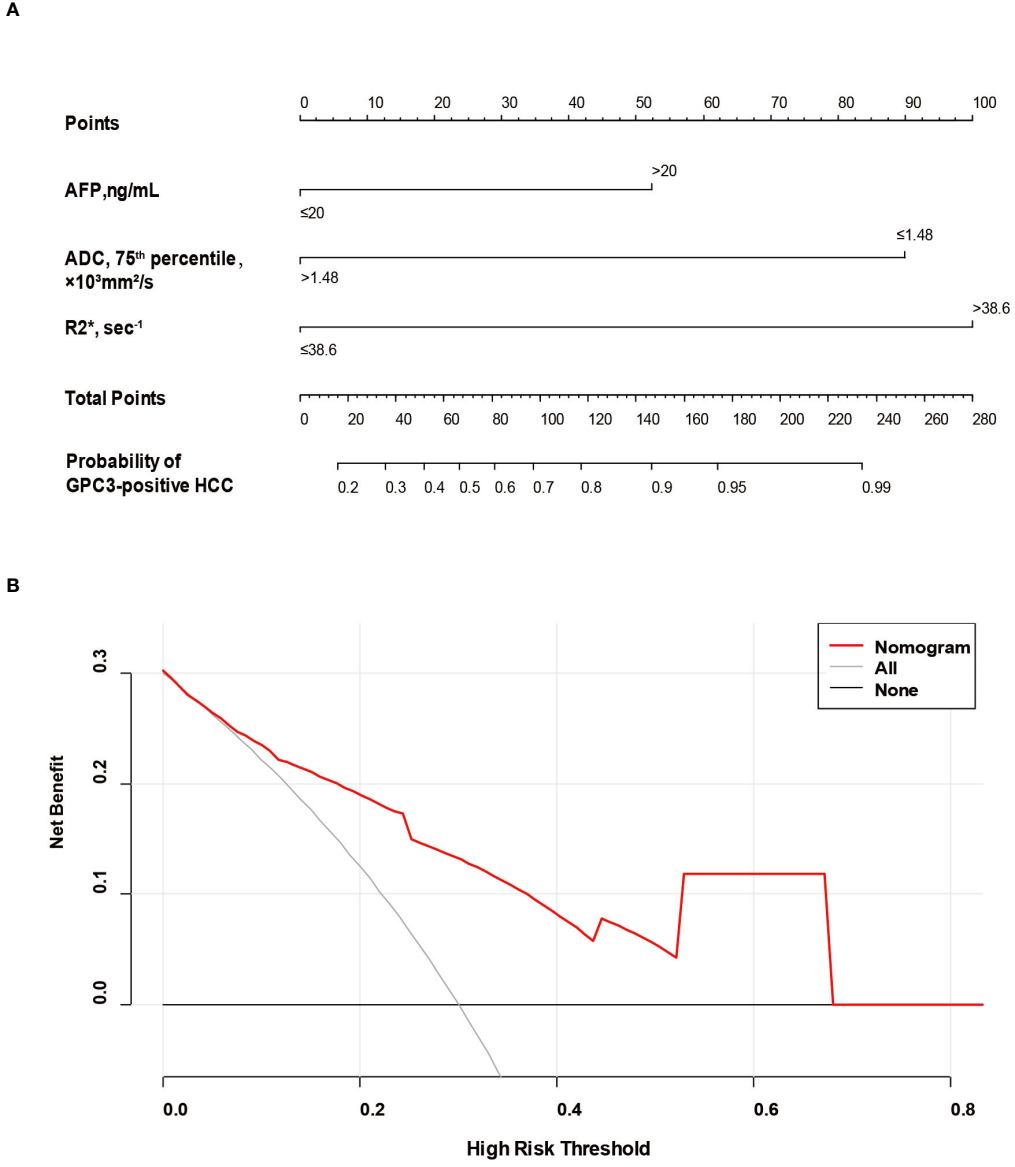
The nomogram and decision curve to predict GPC3-positive HCC. The nomogram **(A)** was developed based on serum AFP levels, 75^th^ percentile ADC value and R2* value. Predictor points are found on an uppermost point scale that corresponds to each variable. On the bottom scale, points for all variables are added and translated into the probability of GPC3-positive HCC. Decision curve **(B)** analysis of the prediction model for external validation set. The X-axis is the probability threshold. Y-axis represents the net benefit, which is calculated by gaining the true positives and deleting the false ones. GPC3, glypican-3; HCC, hepatocellular carcinoma; AFP, alpha-fetoprotein; ADC, apparent diffusion coefficient.

**Figure 5:**
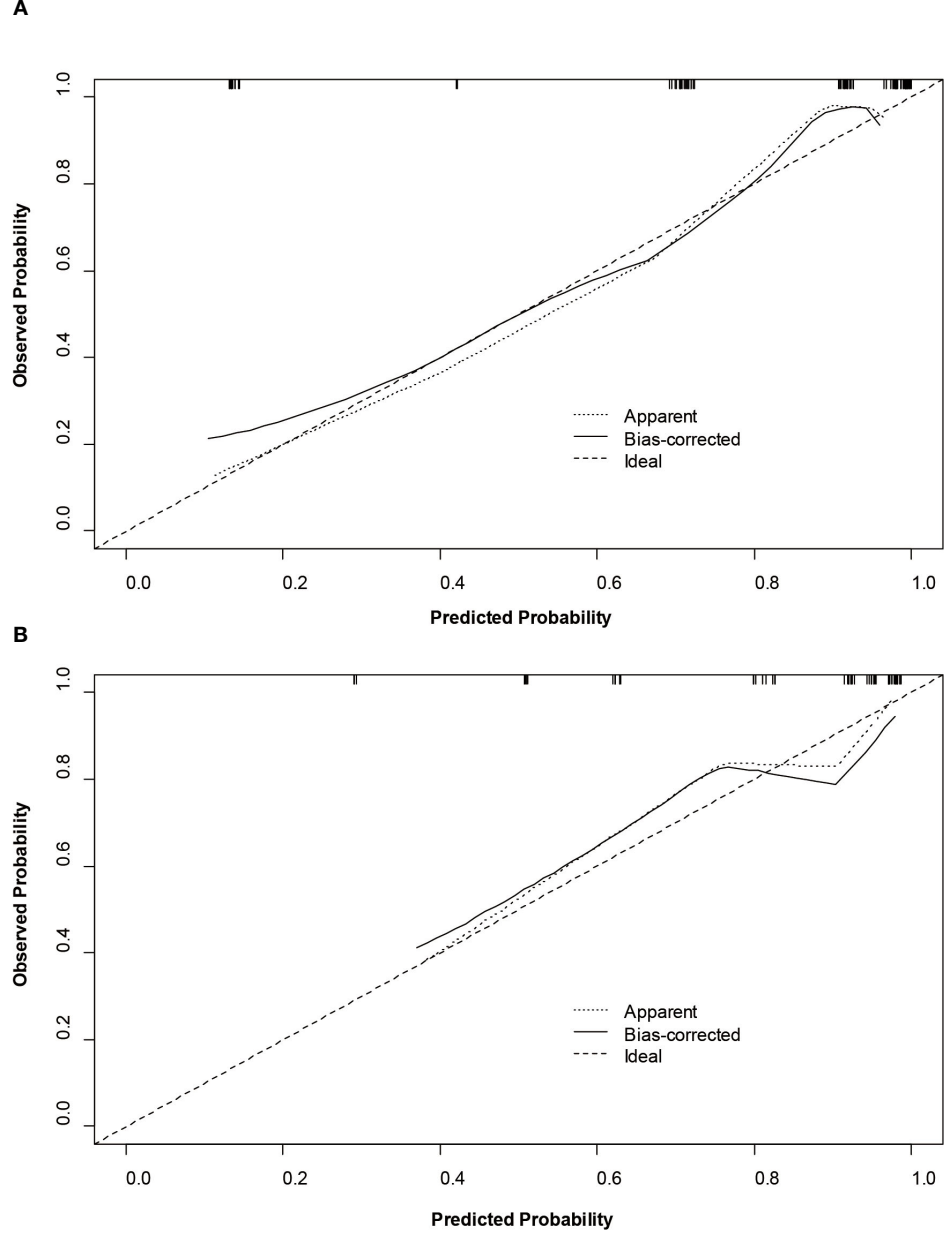
Calibration curves of the nomogram on the training **(A)** and validation **(B)** sets to predict glypican-3 positive hepatocellular carcinoma.

Based on SMOTE-LR model, we construct the nomogram. As can be observed in **Figure 4**, the nomogram and the decision curves confirm the substantial clinical benefits of the prediction model in predicting GPC3-positive HCC. Moreover, the calibration curves demonstrate a good agreement between the predicted and observed probabilities of GPC3-positive HCC for both the training (*P*=0.342) and validation (*P*=0.101) datasets (**Figure 5**).

## 4 Discussion

We developed and validated a noninvasive approach to predict GPC3 expression based on serum AFP levels, R2* and 75^th^ percentile ADC values. With regards to the external validation set, a good diagnostic performance and calibration of GPC3 expression was observed for the prediction model. However, the lower NPV results in the training and validation sets may be partly attributed to the low prevalence of the GPC3-negative HCCs. Nevertheless, the proposed nomogram model was remarkably effective in predicting GPC3 expression.

Previous studies have shown that GPC3 is actively involved in the regulation of HCC tumor growth, and positive expression of GPC3 is associated with poor clinical prognosis in HCC patients. Currently, novel treatments of HCC targeting GPC3 are explored and assessed in *in vitro* and *in vivo* experiments and clinical trials. Such treatments include CAR T cell therapy (29), immunotoxin therapy, increased antitumor activities of glypican-3-specific chimeric antigen receptor-modified T cells by coexpression of a soluble PD1–CH3 fusion protein (30), and GPC3-derived peptide vaccines (31). Therefore, preoperative, noninvasive and precise evaluation of GPC3 expression in HCC is of high significance. In a study by Chen R et al. (26), R2* was shown to provide excellent differentiation between tumors with positive and those with negative GPC3 expression. However, the study was limited from a number of aspects including its small sample size, being single-center, not developing a prediction model and not performing external validation. In addition, some studies have achieved satisfactory results in predicting GPC3 expression in HCC by establishing models using histogram analysis (21) or radiomics (17, 18). However, challenges such as obscure algorithms and complex operations are its main drawbacks in clinical practice. Besides, these single-center studies did not have independent external validation datasets, and the generalizability of the results has not been assessed. That is to say, external test sets are considered essential if the clinical translation of such models is to be guaranteed. Whereas in the present study, good robustness was evaluated by applying the nomogram model on the external validation dataset acquired with different MRI scanners and imaging protocols. Chen Y et al. (15) also developed a nomogram model based on serum AFP and five MRI features; the model was validated in an external dataset. However, all five MRI features were qualitative. The PPV and NPV of the model were relatively low, 0.851 and 0.524 in the training set and 0.794 and 0.400 in the external validation set. Accordingly, the PPV and NPV of these structural radiological features remained controversial. However, the PPV and NPV obtained by constructing the model using multi-parameter quantitative MRI features were 0.869 and 0.615 in the training set and 0.817 and 0.500 in the external validation set. Moreover, the inter-observer agreements were observed to be excellent for the quantitative MRI parameters (ICC = 0.858, 95% CI: 0.731 ~ 0.916 for R2* values, and ICC = 0.882, 95% CI: 0.726 ~ 0.952 for 75^th^ percentile ADC values). Therefore, the model proposed in this study is more accurate and reliable due to its high reproducibility.

According to the obtained results of the present study, the serum AFP levels of > 20 ng/mL, R2* value ≥ 38.6 sec^-1^ and 75^th^ percentile ADC value < 1.48 ×10^3^ mm^2^/s were significantly associated with positive GPC3 expression. In one study, a GPC3-based immunomagnetic fluorescent system (C6/MMSN-GPC3) was proposed by Chu et al. (32). The system was capable of showing the high-specific isolation and instant observation of HCC circulating tumor cells. However, the proposed model is advantageous because GPC3 expression in HCC tumor tissue is directly assessed by MRI exams without the need for peripheral blood and radiation caused by CT scans.

R2* value was also found to be an independent risk factor for GPC3 expression in this study. Currently, the measurement of R2* has been widely used to quantify iron content in patients with liver cirrhosis (33). Chen et al. (26), for example, reported that the significantly higher R2* values in patients with GPC3-positive HCC than those with GPC3-negative HCC is an indication of the increase of the iron content in association with GPC3-positive expression. The finding is consistent with the results obtained in the present study. This can be argued to be due to the overexpression of transferrin receptors on the surface of HCC cells resulting in iron deposition (7, 34). In addition, GPC3 expression was found to be associated with angiogenesis, which tends to occur in HCC-associated microhemorrhages (7, 35). It can in turn contain paramagnetic substances, which can lead to the local magnetic field inhomogeneity resulting in elevated R2*.

What is more, tumor heterogeneity is considered to be an important malignancy feature of HCC. The histogram of the whole tumor volume provides data on multiple parameters. Such data include all voxel distributions in different dimensions, reflecting the heterogeneity of the whole tumor, and are more reliable than the mean value (36, 37). Our study demonstrated that the most commonly used mean ADC value was not significantly different, while median ADC and 75^th^ percentile ADC values were effective predictors of GPC3-positive HCC, of which the 75^th^ percentile ADC value was found to be an independent predictive factor. The 75^th^ percentile ADC value is an ADC value below the 75% region, which indicates that 25% of the maximum value is excluded. This may represent areas of necrosis within the tumor. Therefore, areas of weaker tumor activity may be excluded, resulting in the better representation of the ADC data. In addition, elevated serum AFP levels were observed to be positively correlated with the poor differentiation, microvascular invasion and tumor recurrence (38, 39), which is consistent with the biological behavior of GPC3-positive HCCs. These factors were all considered in the present nomogram model which led to the higher reliability and clinical feasibility in the prediction of GPC3-positive HCC.

In addition to quantitative MRI features, the differential ability of qualitative MRI features of GPC3-positive HCCs were also comprehensively analyzed in the present study. Our results showed a significant difference in peritumoral enhancement in the GPC3-positive group compared to the GPC3-negative group, while the enhancing capsule, and peritumoral hypointensity on HBP were marginally significant. Peritumoral enhancement may represents compensatory hepatic arterial hyperperfusion surrounding the tumor due to portal branch microthrombosis (40). The absence of enhancing capsule and peritumoral hypointensity on HBP suggested the possibility of breakdown of the tumor margin barrier, which is considered to be a risk factor for invasive and metastasis of HCC (41, 42). Previous studies have demonstrated that the above three features correlated with infiltrative appearance. The infiltrative appearance may represent true infiltration of tumor cells into the liver parenchyma, which commonly indicates malignancy with a permeative growth pattern and is associated with macrovascular invasion (43), tumor metastasis and a short survival time (44).

However, it is worth noting that the present study has potential limitations. First, as a retrospective study, only solitary HCCs were selected, which may suggest certain bias in the selection process. The extrapolation of the proposed prediction model to multiple tumors is also hindered by the design of the study. Thus, future studies are encouraged to explore the correlation between MRI features and GPC3 expression in multiple tumors. Second, the small sample size may affect the robustness of the model. Therefore, future studies are suggested to further optimize the prediction model through performing large-scale and multicenter research works. Third, the iron content of the HCC specimens failed to be quantitatively analyzed. Nevertheless, based on the literature, R2* value yield from IDEAL IQ sequence in MRI was proven to be able to accurately quantify the iron deposition in the liver diseases (22, 45, 46). Therefore, the R2* value is expected to be a reliable factor in evaluating the iron content of the HCC in this study. Finally, three-dimension manual ROI segmentation is time- and labor-consuming; therefore, it is essential to develop a user-friendly tool for automatic segmentation to encourage the use of radiomics in a daily radiology reading room.

## 5 Conclusions

An easy-to-use nomogram model was developed and validated for patients with single HCC. The proposed model is capable of accurately predicting GPC3-positive HCC preoperatively based on multiparametric MRI and serum AFP levels. The prediction model is also expected to help identify potential responders to GPC3-targeted immunotherapies and guide personalized treatment decision-making.

## Data availability statement

The original contributions presented in the study are included in the article/supplementary material. Further inquiries can be directed to the corresponding author.

## Ethics statement

The studies involving humans were approved by The first people’s hospital of Zhaoqing. The studies were conducted in accordance with the local legislation and institutional requirements. The ethics committee/ institutional review board waived the requirement of written informed consent for participation from the participants or the participants’ legal guardians/next of kin because of the retrospective nature of the study.

## Author contributions

PL, WL, GQ, JC, and ML conducted the literature search. PL, WL, GQ, YL, ZW, and YZ designed the study. PL, WL, GQ, JC, ZW, and ML collected the data. WL, GQ, JC, ZW, and YZ analyzed the data. All authors verified the data. PL, WL, GQ, JC, YL, ZW, ML and YZ edited the manuscript. PL, QG, J YZ reviewed the manuscript. All authors contributed to the article and approved the submitted version.
